# Hepatic progenitor cells in canine and feline medicine: potential for regenerative strategies

**DOI:** 10.1186/1746-6148-10-137

**Published:** 2014-06-19

**Authors:** Hedwig S Kruitwagen, Bart Spee, Baukje A Schotanus

**Affiliations:** 1 Department of Clinical Sciences of Companion Animals, Faculty of Veterinary Medicine, Utrecht University, Yalelaan 104, 3584 CM, Utrecht, The Netherlands

**Keywords:** Liver, Progenitor cell, Dog, Cat, Regenerative medicine

## Abstract

New curative therapies for severe liver disease are urgently needed in both the human and veterinary clinic. It is important to find new treatment modalities which aim to compensate for the loss of parenchymal tissue and to repopulate the liver with healthy hepatocytes. A prime focus in regenerative medicine of the liver is the use of adult liver stem cells, or hepatic progenitor cells (HPCs), for functional recovery of liver disease. This review describes recent developments in HPC research in dog and cat and compares these findings to experimental rodent studies and human pathology. Specifically, the role of HPCs in liver regeneration, key components of the HPC niche, and HPC activation in specific types of canine and feline liver disease will be reviewed. Finally, the potential applications of HPCs in regenerative medicine of the liver are discussed and a potential role is suggested for dogs as first target species for HPC-based trials.

## Introduction

Regenerative medicine is a rapidly developing field in which diseased tissues are restored or regenerated. This interdisciplinary field converges biomedical research, technology and clinical care, and is based on the concept of employing intrinsic repair mechanisms within the tissue itself. A hallmark of regenerative medicine is the clinical use of stem cells, either by manipulation of endogenous progenitor populations *in situ*, or by transplantation of stem cells (autologous or allogeneic). Recent developments in human stem cell therapy are highly visible and it appears that this phenomenon is now also entering the veterinary clinic. In April 2013, *Nature* published a report in its news section on the growing use of stem cells in veterinary medicine. Although popularity has increased, the efficacy of many stem cell therapies is often unproven. New FDA regulations in the USA are pending and if stem cells are defined as a drug, application as a new treatment modality requires evidence-based veterinary medicine [1].

**Figure 1:**
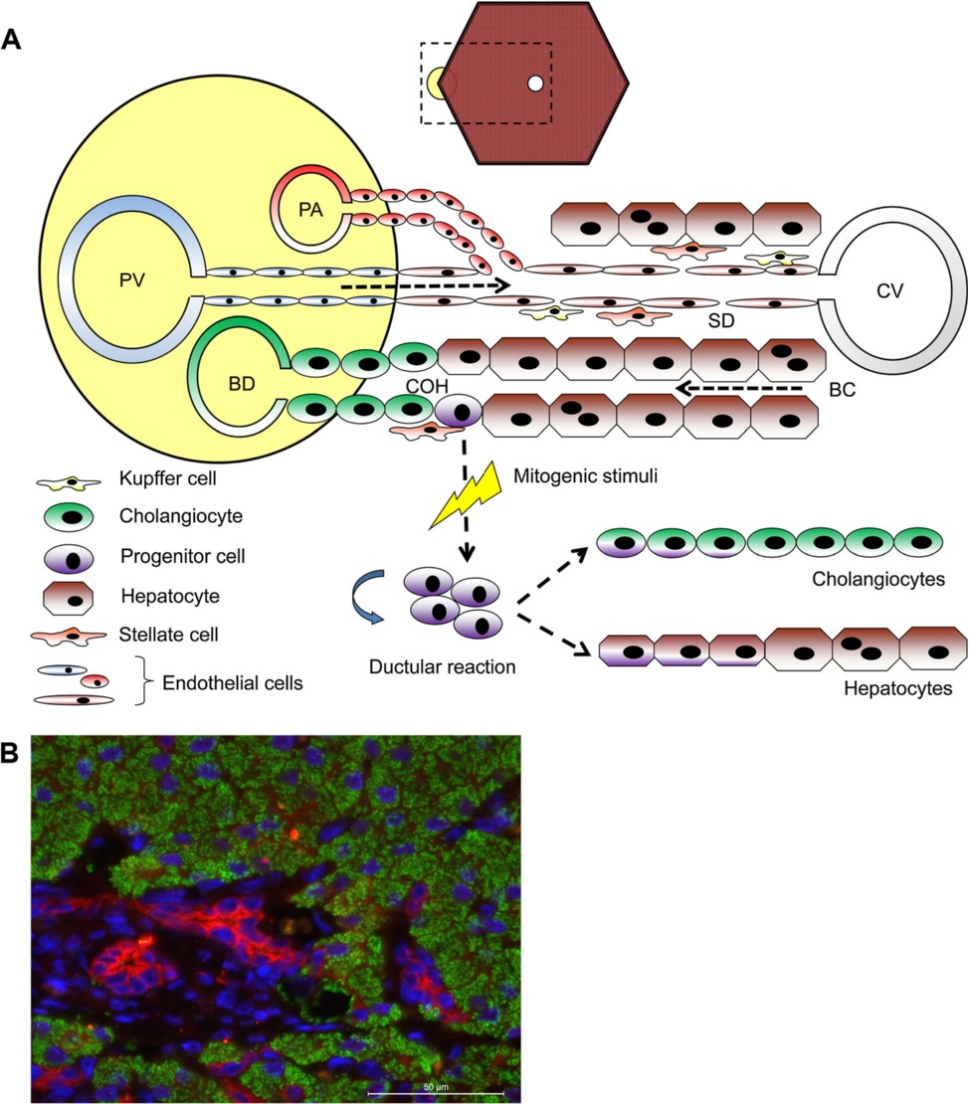
**Anatomical location and differentiation capability of hepatic progenitor cells. A**. Schematic representation of the anatomical location of the hepatic progenitor cell (HPC) in the canal of Hering. Upon activation the normally quiescent HPCs will proliferate. Depending on the disease and the concurrent changes in microenvironment HPCs will differentiate into either hepatocytes or cholangiocytes. PV: portal vein; BD; bile duct; PA: portal artery; COH: canal of Hering; SD: space of Disse; BC: bile canaliculus; CV: central vein **B**. Immunofluorescent double staining of panCK (red) and HepPar-1 (green) with a nuclear counterstaining (DAPI, blue) of a liver section of canine chronic hepatitis. Differentiation into hepatocytes can be observed where the ductular reaction enters the parenchyma as the intermediate hepatocytes lose panCK immunoreactivity and become positive for HepPar-1 [7].

Regenerative strategies in the liver seem redundant, as adult hepatocytes are widely known for their large regenerative capacity. However, developments in the field of hepatology make clear that in severe or chronic ongoing liver disease, regeneration by hepatocyte replication is failing or absent [2]. In these specific circumstances liver-specific stem cells, or hepatic progenitor cells (HPCs), become activated and attempt to repopulate the liver. HPCs are a reserve compartment of adult stem/progenitor cells that reside within the liver and are found in rodents, humans, dogs and cats [3-7]. HPC activation in a diseased liver section is described as ‘ductular reaction’ or ‘bile duct proliferation’ in a histology report [8,9]. Diagnostically, it indicates severe liver disease. In addition, the presence of progenitor cell markers in hepatocellular carcinoma (HCC) is an indicator of malignancy in humans as well as dogs [10-12]. Conversely, HPCs hold potential as a therapeutic target since they are committed liver stem cells, show self-renewal capacity and can differentiate into hepatocytes and cholangiocytes (Figure 1) [13]. Literature on HPCs focuses on mouse, rat, and human. There are few publications on canine HPCs and even fewer on cat or other species and it is clear that the HPC response is often referred to as ‘bile duct proliferation’ when observed in liver histological sections [8,14]. In this terminology there is no suggestion of the presence and activation of stem cells, implying that the presence of HPCs in the liver of dogs and cats is not widely recognized and that there is no consensus on terminology in veterinary pathology. An attempt to achieve this consensus in clinical and histological diagnosis of liver disease has been made by the WSAVA Liver Standardization Group.

In this review, we will provide an overview of the role of HPCs in liver regeneration and will address the most important cellular and stromal players in HPC biology. Although current knowledge about HPCs stems primarily from experimental rodent and clinical human studies, we will review available literature on HPCs in canine and feline liver regeneration, and support these with recent data from our own research. To conclude, we will discuss the possible use of HPCs for clinical purposes in veterinary regenerative medicine and for future research needs.

### The role of HPCs in liver regeneration

Seventy percent of the liver consists of mature hepatocytes located in the parenchyma. These adult hepatocytes are normally quiescent, but enter the cell cycle when the liver is damaged. They can restore liver function by compensatory hyperplasia, an efficient and well-orchestrated physiological response [15]. The large replicative potential has designated hepatocytes as a stem cell of the liver in the past [16], but their lack of differentiation potential does not render them true stem cells [17]. This process of liver regeneration has been thoroughly investigated by using the partial hepatectomy (PHx) model in rodents as well as in dogs, and has revealed the involvement of a plethora of growth factors and cytokines [2,18-21]. Previous work by our group demonstrates that in canine liver disease the primary molecular pathways associated with liver regeneration (e.g. the hepatocyte growth factor (HGF) signaling pathway) are highly comparable with those in rodents and humans [22-24]. For the cat, the underlying molecular mechanisms of disease and regeneration have not been described.

Upon acute severe or chronic hepatic injury, hepatocyte replication is impaired or exhausted. This impairment in hepatocyte replication is linked to an increase in HPC activation [2]. For example, in biopsies of human patients with severe acute liver damage it was shown that more than 50 percent hepatocyte loss results in a lower proliferative activity of the remaining hepatocytes, when compared with less severe hepatic injuries. This was associated with a pronounced HPC response, and positively correlated with symptoms of liver failure [25]. Hepatocyte senescence occurs in chronic liver disease, which is characterized by increased p21 expression (cell cycle inhibitor) and shortened telomeres in the hepatocytes [26,27]. A report from Liu et al. showed that when hepatocytes from a cirrhotic donor rat were transplanted into a non-cirrhotic host liver, the cells engrafted but showed decreased metabolic function and delayed proliferation due to replicative senescence [28]. This phenomenon of hepatocyte senescence was also observed in a mouse model of fatty liver disease and a marked progenitor cell response was observed in the affected animals when compared to their wild type controls [29].

**Figure 2:**
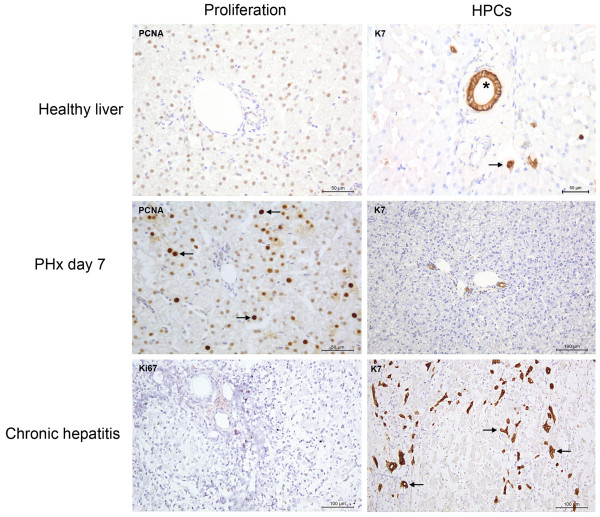
**The first and second line of defense in canine liver regeneration.** In liver sections proliferation is visualized by PCNA or Ki67 immunohistochemistry. K7 was used as a marker for hepatic progenitor cells. In healthy liver, both hepatocytes and HPCs are quiescent, indicated by a few hepatocytes that stain for PCNA and only a few K7 positive cells close to the portal area (indicated with arrow, asterisk indicates bile duct). After partial hepatectomy (PHx), liver regeneration occurs through hepatocyte proliferation (many PCNA positive hepatocytes indicated by arrows) but the HPC remains quiescent (few K7 positive cells). In chronic hepatitis the proliferative capacity of hepatocytes is exhausted indicated by a few Ki67 positive hepatocytes and a prominent ductular reaction (K7 positive, indicated by arrows) [6,30].

Hepatocyte senescence in chronic liver disease has not been investigated in the dog and cat. However, immunohistochemical stainings for PCNA or Ki67 in various canine liver diseases show prominent proliferation of hepatocytes after experimental PHx and mild acute hepatitis, with moderate proliferation in chronic hepatitis. Conversely, HPC response was pronounced in chronic hepatitis, moderate in mild acute hepatitis and non-existent after PHx (Figure 2) [6,30]. The response pattern of HPCs to various types of liver disease in the dog appears to be comparable to human pathology and rodent experimental findings, and recent studies suggest a similar comparison for feline HPC response [6,7,31], [Unpublished observations section: Valtolina et al.]. In all species, HPC response correlates with the severity of disease and is localized at the site of disease activity [6,25,32,33]. The current consensus is that the HPC pool is a reserve compartment in the liver that contributes to regeneration when hepatocytes do not replicate sufficiently to restore liver mass and function.

### The hepatic progenitor cell

HPCs are present in healthy adult liver tissue and can be found in small numbers in the Canal of Hering, the smallest ramifications of the intrahepatic biliary tree, which connect to the intralobular canaliculi. These structures are located close to the portal area and are lined by both cholangiocytes and hepatocytes [34]. This is the most commonly described HPC niche, although there is still debate about the exact origin of the HPC. A number of studies state a possible biliary origin of HPCs [35-37]; other studies in humans describe extrahepatic peribiliary glands as the prime location for HPCs [38,39]; and a few publications even speculate on a hematopoietic origin of HPCs, which is also highly debated [40-44]. For this review we assume an HPC niche within the Canal of Hering, as described in mouse, rat, human, and dog [3,4,7,45]. HPCs can be histologically characterized by a combination of their specific morphology upon activation (ductular reaction, DR) and by marker expression. Many classic HPC-markers, such as keratin (K)7 and K19, have a shared expression with cholangiocytes, which underlines the significance of combining the interpretation of marker expression with histological evaluation. Other reported markers include CD133 and EpCAM, which are also expressed in other stem cells such as hematopoietic or embryonic stem cells (for a review, see [46]). HPCs are epithelial cells that can display mesenchymal characteristics, depending on their activation status (e.g. need for migration capacity). This is reflected in the expression of CD29 (integrin β1) and CD44 (hyaluronic acid receptor and co-receptor for hepatocyte growth factor), proteins involved in cell-matrix interactions and potentially critical for cell migration. When reviewing HPC marker expression, interspecies differences emerge. Therefore, it is necessary to evaluate appropriate markers in the species of interest and, in rodents, to consider the model used [47]. In Table 1 we provide an overview of available literature on HPC markers in mouse, rat, human, dog, and cat. It is important to take into account that the HPC niche can be dynamic during its various states of quiescence, proliferation and differentiation, which is reflected by marker expression. Some markers (such as CD133 and Lgr5) are expressed by only a subset of cells or only upon activation [48,49].

**Table 1:** Comparison of HPC marker expression across species

**Marker**	**Mouse**	**Rat**	**Cat**	**Dog**	**Human**
A6	[3,50]				
ABCG2/BCRP1	[47]	[47]		[7]	[7,48]
AFP		[47,51,52]		[53]	[48,54-56]
Alb	[57]				[54,55,58]
Dlk/Pref-1	[59]	[47]			
c-kit		[60]			[48,56]
CD24	[50]				
CD29		[51]		[53]	[54]
CD34		[60]			
CD44	[49]	[51]		[53]	[48,54,55]
CD45		[60]			
CD73					[54]
CD90		[52,60]			[54]
CD133/PROM1	[49,57,59,61]	[51]		[53]	[48]
CLDN3					[55]
chrom-A					[32,33]
EpCAM	[50,57,59]	[45,51]			[55,62]
FN14	[59]	[51]		[53]	
GPC3		[52]			
Hedgehog proteins				Schotanus (unpublished data)	[55]
HNF4α		[45]		[53]	
ICAM1					[55]
K7	[36,57]	[32,63]	[31]	[6,7,53]	[6,7,25,32,33,48,64,65]
K8					[33,54]
K18					[33,54]
K19	[3,37,57,59]	[32,45,51,52,63,66]		[6,53]	[6,25,32,33,48,55,56,58,64,67]
Lgr5	[49]				
MPK		[47]			
NCAM					[32,48,55]
NES					[54]
Nope	[50]				
OPN	[37]			[53]	
OV6		[32,45,66]			[32,33]
Sca1	[59]				
SOX9	[36,37,49,59,61]			[53]	
vimentin					[54]

References are indicated per marker per species. Expression was measured at mRNA and/or protein level and was reported for adult liver.

### Cells, signals and stroma in the HPC niche

An essential feature of stem cell biology is the niche, or micro-environment, in which stem cells reside. It consists of neighboring cells, extracellular matrix (ECM) components and soluble and bound growth factors and cytokines that govern self-renewal and maturation/differentiation status [68]. The composition of the HPC niche is well defined and adapts during specific types of liver disease [44,69]. A number of cellular niche components have been described, and below we discuss the hepatic stellate cell, the macrophage and the ECM.

### Hepatic stellate cells

Hepatic stellate cells (HSCs, or previously called Ito cells) are found in the space of Disse and can transform into myofibroblasts upon injury-induced activation. Quiescent HSCs are important in vitamin A storage (mainly as retinol-containing lipid droplets) and function as liver resident antigen presenting cells [70,71]. Activated HSCs produce ECM components such as collagen and are the main contributors to fibrosis development in chronic liver disease [72]. Interestingly, HSCs are also an essential mediator of the HPC response and the primary source of HGF, which stimulates hepatocyte and HPC proliferation and liver regeneration [73,74]. HSCs may also play a role in directing the differentiation of HPCs, and co-culture studies of HSCs and HPC-like cells indicate that this is probably mediated by both soluble and membrane-bound factors or matrix components [75].

### Macrophages

Macrophages in the liver are a second important niche component. Macrophages are activated upon hepatocyte damage and are integral to the local immune response [76]. Cytokines (e.g. TWEAK) produced by this inflammatory cell can modulate HPC behavior over large distances in the tissue [77,78]. HPC migration through the parenchyma was significantly decreased in mice depleted for macrophages with clodronate and subsequently subjected to liver injury [79]. Boulter et al. corroborated this finding by reporting a pivotal role of both activated myofibroblasts and macrophages in murine HPC differentiation. Mediated by Wnt and Notch signaling, respectively, macrophages are involved in the specification of hepatocyte differentiation upon hepatocellular injury and myofibroblasts promote biliary differentiation of HPCs [80]. These data support previous studies on the involvement of Wnt and Notch signaling in human clinical HPC activation. In human samples of acute hepatitis, a parenchymal liver disease, the activated HPC niche showed increased Wnt signaling. Active Notch signaling in the activated HPC niche was mainly observed in biliary-type diseases [48].

### Extracellular matrix

A third critical component of the HPC niche is the extracellular matrix (ECM) and its specific composition. In particular, laminin has been shown in both mouse models and human fibrotic liver disease to play an important role in HPC biology. A laminin matrix develops in many liver diseases and consistently surrounds the ductular reaction. The deposition and remodeling of laminin is required for HPC proliferation and migration and it maintains the undifferentiated state of the HPCs. It is only when the HPCs ‘escape’ from the laminin matrix and enter the parenchyma that differentiation occurs [44,81,82]. HPCs express markers such as CD29 and CD44, clearly indicating that they have the molecular make-up to communicate with their ECM [59,83]. Interestingly, ECM remodeling is modified by HSCs and macrophages through expression of matrix metalloproteinases (MMPs) and tissue inhibitors of metalloproteinases (TIMPs), and is associated with the extent of ductular reaction and fibrosis [76,84]. Several studies suggest a direct relation of HPCs with increased fibrosis development and remodeling [85,86].

To date, there are only a few publications on HPC niche components in dog. An immunohistochemistry study evaluated the inflammatory infiltrate and fibrosis in samples of canine chronic hepatitis, and recorded an increased amount of ‘bile duct proliferation’ in cases with marked inflammation and more advanced stages of fibrosis. A positive correlation was found between the stage of fibrosis and the number of myofibroblasts and bile duct proliferation [87]. The location and characteristics of quiescent canine HSCs and portal myofibroblasts were characterized in healthy liver. HSCs were found in the space of Disse as previously described for other species [88]. A subsequent study focused on samples of canine chronic hepatitis and lobular dissecting hepatitis and reported a positive correlation between the presence of tenascin-C, a specific component of ECM, and stage of fibrosis, degree of inflammation and the number of K7 positive cells [89]. These findings confirm HPC activation upon severe liver disease in the dog and suggest an association with stellate cells and/or myofibroblasts, but do not exactly specify the HPC niche components.

**Figure 3:**
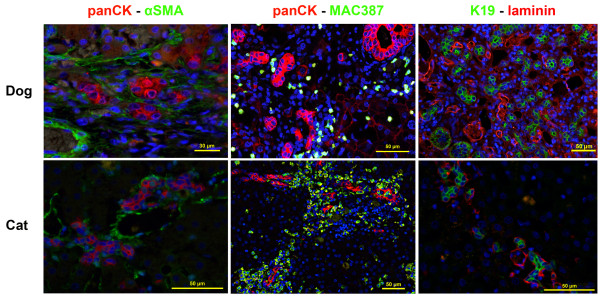
**Cellular and stromal components of an activated hepatic progenitor cell niche in dog and cat.** Immunofluorescent double stainings of liver sections of canine chronic hepatitis and feline chronic neutrophilic cholangitis. PanCK or K19 was used as a marker for HPCs, activated stellate cells are visualized using αSMA staining and macrophages using MAC387 staining. Nuclei were counterstained with DAPI (blue). In canine and feline liver disease there is clear colocalization of activated HPCs with hepatic stellate cells, macrophages and laminin. [53, Unpublished observations section: Valtolina et al.].

A publication on the relation between HPCs, HSCs, fibrosis and disease severity in healthy and diseased liver samples describes the presence of activated HSCs in close vicinity to the ductular reaction in all types of liver disease studied. In liver disease with fibrosis, HPC activation was most pronounced and both HPCs and HSCs localized to the primary site of injury [6]. This was substantiated by a second study, using immunofluorescent double stainings to evaluate HPCs and their niche in different types of liver disease (Figure 3). Activated stellate cells, characterized by positive alpha-smooth muscle actin (αSMA), were predominantly present in fibrotic liver diseases, such as lobular dissecting hepatitis and chronic hepatitis. HSCs colocalized with the prominent ductular reaction and this colocalization was also seen for laminin at the site of disease activity where it consistently surrounded the ductular reaction. Total macrophage numbers were significantly increased in chronic hepatitis and lobular dissecting hepatitis. Although macrophages were identified throughout the parenchyma, they appeared to cluster at the injury site; periportal in acute hepatitis and in the fibrotic septa in chronic hepatitis [53].

To our knowledge no literature available for cats on the interaction or co-occurrence of HPCs, HSCs, macrophages and/or ECM. In light of the similar presence of HPCs in liver disease in cats, one would also expect a highly activated and comparable HPC niche in these animals [14,31]. Recent unpublished data indeed show similar involvement of HSCs, macrophages and laminin in the feline HPC niche (Figure 3) [Unpublished observations section: Valtolina et al.].

### HPC activation in different types of liver disease in man, dog and cat

**Figure 4:**
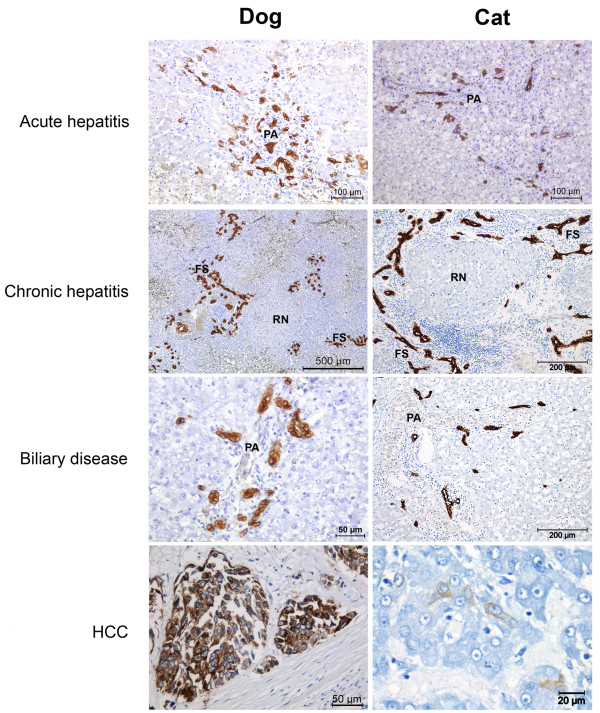
**Hepatic progenitor cell activation in liver disease in dog and cat.** K19 immunohistochemistry of liver sections from different types of liver disease in dog and cat. HPCs are activated in acute and chronic hepatitis and in biliary disease. The extent and location of the ductular reaction depends on type and severity of disease. Canine extrahepatic cholestasis and feline neutrophilic cholangitis were selected as representative biliary diseases. The lower panel shows K19 positive hepatocellular carcinoma in dog and cat. [6,11, Unpublished observations section: Valtolina et al., Van Sprundel et al.].

In the following section, the HPC response is described as it occurs in various forms of hepatitis, biliary disease and liver tumors. Figure 4 shows a representative selection of diseased canine and feline liver sections stained for K19.

### Acute hepatitis

In human hepatology, severe acute parenchymal liver failure is most often caused by viral infections (e.g. hepatitis A, B, E) and ingestion of toxic substances (e.g. acetaminophen, Amanitum mushrooms) [90,91]. Massive hepatocyte loss triggers an HPC response, and is most apparent in human subjects suffering from acute submassive necrosis [25,64]. This response will rapidly develop and already after 24 hours a prominent ductular reaction can be observed. Proliferation is followed by differentiation, during which the ductular reactions give rise to ‘hepatocyte-like cells’ (also identified as intermediate hepatocytes) that spread into the parenchyma [32].

In the dog and cat, acute liver injury most often presents as (mild) acute hepatitis and is characterized by inflammation and apoptosis/necrosis. Fulminant hepatitis is rarely diagnosed in the veterinary clinic. Etiology is not always known but numerous causative agents have been described. Similar to human hepatology, viral infections can cause acute hepatitis (e.g. canine adenovirus I, canine or feline herpesvirus) and ingestion of toxic substances (iatrogenic or accidental) can result in considerable hepatocellular damage (e.g. Amanitum mushrooms, Cyanophyceae algae, acetaminophen, and benzodiazepines) [9]. The involvement of the HPC compartment in canine and feline acute hepatitis has been described in only very few studies. For canines, a ductular reaction has been observed localized to the site of injury (primarily periportal in acute hepatitis), accompanied by intermediate cells (recognized among others by submembranous K7 staining), suggesting early differentiation [7]. In addition, colocalization of activated HPCs and HSCs has been observed [6]. Ijzer et al. published the only paper specifically describing HPC behavior in liver disease of six cats with acute or fulminant hepatitis. In the periportal areas, there was evidence of an extensive ductular reaction, branching into the parenchyma, containing mitotic figures [31].

### Chronic hepatitis

In humans, chronic hepatitis results in morbidity and mortality world-wide. Important causes are viral infections (e.g. hepatitis C), alcohol abuse, and autoimmune disease [92]. In human chronic hepatitis, the HPC compartment is activated when hepatocyte replication becomes exhausted. A ductular reaction develops and expands with disease severity [33,65,67].

In veterinary medicine, chronic hepatitis is seen predominantly in dogs and infrequently in cats [93,94]. Fibrosis is the histological hallmark and is accompanied by inflammation and hepatocyte apoptosis/necrosis. Regeneration will occur to some extent; in cirrhosis this is represented by hyperplastic nodules of newly formed hepatocytes which emerge between the fibrotic septa [9]. HPCs and their niche are activated and a clear ductular reaction develops at the site of disease activity, which is usually in and adjacent to the fibrotic septa. HSCs are also strongly activated, differentiate into myofibroblasts, and are found at the site of fibrosis surrounding the activated HPCs [6,7]. Chronic hepatitis in dogs is perhaps best characterized as a degenerative process with unsuccessful regenerative attempts in most cases.

### Fatty liver disease

Human non-alcoholic steatohepatitis (NASH) and fatty liver disease ((NA)FLD) are increasingly common hepatic disorders associated with obesity and insulin-resistance [95]. Storage of large quantities of fat and subsequent inflammation can ultimately result in liver fibrosis, cirrhosis and HCC. Human FLD is associated with increased oxidative stress and inhibition of hepatocyte replication. A strong HPC response is observed, which correlates with disease severity and fibrosis [96]. A recent study by Nobili et al. showed similar results in pediatric NASH and NAFLD and revealed adipokine signaling in activated HPCs, suggesting an active (or reactive) role in the steatosis process [97].

In cats, one of the most common hepatic parenchymal diseases is hepatic lipidosis, a fat storage disease. Hepatocytes accumulate fat vacuoles, microscopically appreciated as micro- or macrovesicular steatosis [98,99]. In sections of feline hepatic lipidosis a ductular reaction was observed, which extended into the periportal parenchyma and was associated with intermediate hepatocytes [31]. Awareness about the existence of feline HPCs during hepatic lipidosis and the appropriate terminology describing their histological appearance are currently lacking [98]. Since they possibly share a common etiology of metabolic dysfunction, the histological similarity of feline lipidosis to human NASH and NAFLD at the tissue level is currently under investigation. It appears that these fat-storing hepatic diseases have a comparable histopathological reaction pattern to inflammation and fibrosis [Unpublished observations section: Valtolina et al.].

### Canine lobular dissecting hepatitis

Lobular dissecting hepatitis (LDH) is unique only to dogs, and displays extraordinary clinical behavior and histology. LDH has an acute disease progression but is histologically characterized as a chronic hepatitis, due to the occurrence of extensive fibrosis. Interestingly, in LDH a massive and unrivalled expansion of the HPC pool is seen dispersed throughout the parenchyma [6,9,89]. When the HPC niche was studied in detail using laser-microdissection, expression of self-renewal and progenitor markers was present, but markers of hepatocyte differentiation were absent. This is indicative of a strong proliferative response that is not followed by appropriate differentiation. Recent work showed that pre-existent liver fibrosis impaired liver regeneration upon partial hepatectomy in mice. Impaired liver regeneration was associated with increased HPC proliferation and *de novo* fibrogenesis. Interestingly, suppression of the HPC response attenuated fibrogenesis and restored regeneration by mature hepatocytes [100]. Perhaps in LDH the high amount of fibrosis somehow interferes with the maturation/differentiation of the cells in the ductular reaction, suggesting a disturbed niche biology [53]. Further research is needed to clarify the potential contribution of HPCs to fibrosis progression and their potential negative contribution to liver regeneration. LDH could be a very interesting disease to investigate this phenomenon [85,86].

### Biliary disease

In human biliary disease, a local regenerative response results in bile duct proliferation, most probably comprising of both HPC activation and proliferation of pre-existing bile duct cells [32]. As markers for HPCs often overlap with cholangiocyte markers, it can be challenging to ascertain the specific origin of newly formed bile ducts. However, in the case of biliary cirrhosis specific stainings suggested HPCs to be the cell of origin to repopulate and regenerate injured bile ducts [101]. Canine biliary diseases include extrahepatic cholestasis and destructive cholangitis. These diseases present with an activated HPC niche but are not often diagnosed [53,102]. In felines, biliary disease is frequently seen, most commonly lymphocytic and neutrophilic cholangitis, and are associated with inflammatory cell infiltrates [94,103,104]. Lymphocytic cholangitis is a chronic disease that results in portal fibrosis and bile duct proliferation [14,102]. In a large cohort of feline liver biopsies Gagne et al. observed bile duct proliferation in 26 out of 27 cats with lymphocytic cholangitis. Both the extent of bile duct proliferation and the degree of fibrosis were positively correlated with the severity of the inflammatory infiltrate. In 10 out of 11 cats with neutrophilic cholangitis, an acute biliary disease, bile duct proliferation was observed [94]. Similar to humans and dogs it is likely that bile duct proliferation in cats involves both cholangiocytes and HPCs.

### Liver tumors

An emerging research area focuses on the association between HPCs and liver tumors, both in man and dog. This association is plausible, as HPCs have self-renewal capacity and migratory potential, which is required for invasion and metastasis [105]. However, the presence of HPC features within a liver tumor can be explained by more than one theory. First, HPCs are described as a possible cell of origin for hepatocellular carcinoma (HCC) and cholangiolocellular carcinoma (CLC, a specific type of cholangiocarcinoma), although no one has yet directly shown this lineage relationship [10,106-109]. Second, the presence of HPC markers in HCC is compatible with the possible dedifferentiation of resident hepatocytes that undergo malignant transformation, resulting in the expression of immature markers like K19 on HCCs [106,110].

There is clinical evidence that expression of HPC markers in human HCC is a negative prognostic indicator, as these tumors show a higher recurrence rate and shortened patient survival [10,111]. In dogs, the presence of progenitor (K19) and malignancy (glypican-3) markers was evaluated immunohistochemically, and related to a histological grade and a staging score (including local or distant metastasis). The occurrence of K19 positive HCCs was 12%, which resembles the prevalence in humans. This K19 positive subset was poorly differentiated and more likely to metastasize, suggesting that K19 may be a malignancy marker in canine HCC [11]. However, for both dog and human it is still unclear whether HPCs are the cell of origin in these types of liver cancer. For liver tumors in cats, an association with HPC characteristics is under investigation by our group [Unpublished observations section: Van Sprundel et al.]. Patnaik et al. demonstrated in a retrospective study of 47 feline liver tumors that the majority of neoplasms were epithelial and primarily of biliary origin [112]. Further research is required to understand whether HPC markers are a prognostic indicator in feline liver tumors.

Ultimately, while further studies are required to reach a definitive answer on the cellular origin of liver tumors, the association between HPC markers and malignancy is now widely acknowledged.

### HPCs in regenerative medicine

**Figure 5:**
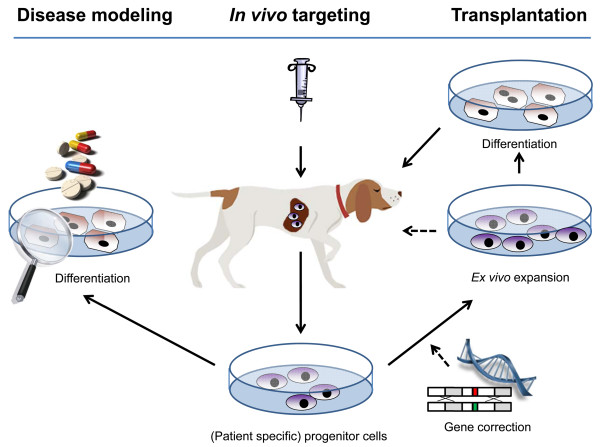
**Application of hepatic progenitor cells in regenerative medicine.** Schematic representation of the potential use of hepatic progenitor cells in veterinary medicine. *In vivo* targeting of a patient’s endogenous HPC population with small molecules would be the most elegant approach. Another option is *ex vivo* expansion of HPCs in culture, potential differentiation into hepatocytes and subsequent use in clinical cell transplantation. Upon differentiation HPCs can also be used for disease modeling. For example, by culturing HPCs from a patient with an inborn error of metabolism it is possible to study mechanisms of disease and to perform drug discovery screens.

For severe parenchymal or biliary liver diseases, definitive and curative treatment options are currently lacking in both human and veterinary medicine. In humans, the final treatment option is a liver transplantation, but many patients die while on the waiting list (for actual data on US organ transplants see UNOS website [113]). Moreover, not all grafts remain viable after transplantation (e.g. due to rejection), warranting extensive immunosuppression or a second transplantation if possible. In dog and cat, liver transplantation is not performed. Since the etiology of liver disease is often not known, current therapy in veterinary medicine is restricted to symptomatic treatment and the use of corticoids [114-117]. To be able to improve patient survival and disease outcome, new curative therapies for advanced liver disease are required. Hepatocyte transplantations have been studied most extensively and have been performed in human patients with metabolic liver disease [118-120]. The use of hepatocytes does, however, not solve the problem of donor-shortage. Additionally, hepatocytes cannot be expanded to reach sufficient numbers for transplantation [121] which also inhibits the establishment of cell banks. The development of ’humanized livers’, where murine or porcine host livers are used as an *in vivo* bioreactor to grow (human) hepatocytes, are potential ways to bypass this problem [122-124], but further research is needed to explore its potential for therapeutic use. Especially for veterinary medicine this approach could raise ethical questions. HPC-based treatment modalities could avoid the problems encountered when using hepatocytes for transplantation. HPCs can self-renew, generating a stable pool of progenitors, and can differentiate into newly generated hepatocytes or cholangiocytes which restore liver function [13,125]. There are two regenerative strategies that could be employed in the (veterinary) clinical use of HPCs and we will briefly discuss them in the light of previous studies (Figure 5).

The first option would be to target a patient’s own HPC population *in vivo* by specific drugs or small molecules. This elegant approach is quick, minimally invasive, does not carry risk of rejection, and has the potential to be relatively cost-effective. The goal would be to activate a patient’s own HPC pool and to boost proliferation and/or differentiation depending on the type of liver disease. A prerequisite is that the essential signals required to mount an HPC response are known, and that these signals are specific to HPCs and do not, for example, activate HSCs and cause excessive fibrosis. Additionally, one needs to consider that overstimulation of the HPC pool might have unexpected and undesirable side effects. HPCs have the capacity of regenerating the liver but in many diseases this is too little and too late. Possibly in these cases specific pathological or molecular characteristics somehow interfere with HPC proliferation or differentiation. Therefore, any signal that is found to benefit the HPC response must be reviewed in a clinical and disease-specific perspective. This highly promising but very challenging approach is currently unexplored in all species. Once these signals are unraveled this approach may become a primary focus for the development of new hepatic regenerative treatments.

The second option is to use differentiated HPCs as a cell source for transplantation, either autologous or allogeneic. Technically it is possible to harvest autologous HPCs from a liver biopsy, expand them in culture and differentiate them into hepatocytes for transplantation purposes. In case of inherited metabolic disease, gene correction could be applied before transplantation. HPCs can be cultured *in vitro* upon isolation from primary canine liver tissue as shown by Arends et al. [126]. Using a plate-and-wait method, they were able to grow colonies of canine HPCs from the non-parenchymal fraction of a digested liver sample within a few weeks. Unfortunately, in cases of urgent clinical needs, this culture method as an autologous source for transplantation would not be feasible. In chronic cases, however, this would be an option and would circumvent rejection issues. Optimization of culture conditions of primary HPCs is needed in addition to characterization of cells in culture, most importantly, self-renewal and differentiation capacity and stability. A promising recent development is the discovery of Lgr5 positive cells in injured mouse livers that can be FACS sorted or isolated as ‘ducts’ and form organoids upon 3D culturing [49,127]. These cells rapidly expand, have the capacity to differentiate into hepatocytes, and can be kept in culture for more than a year, while maintaining their genomic integrity. An important caveat in clinical HPC transplantation are the costs associated with the expansion of HPCs in culture. In veterinary medicine this must be balanced against the amount a pet owner is willing to pay for treatment. The costs will be highly influenced by the number of patients that could benefit from a new therapy [128]. In a UK study, the prevalence of chronic hepatitis in a dog population from first opinion practices was 12% [129], supporting an economical niche to develop new therapeutics for veterinary liver disease. The fact that treatment of dogs may serve as pre-clinical studies for human drug development could provide an economically interesting approach for pharmaceutical industries. The predicted doubling frequency of end stage liver disease in man worldwide shows the medical and economic relevance to design new therapies for human liver disease [113]. As stem cell-based therapies are being developed for multiple organs and diseases, advances are likely to be made in the near future [128].

When planning the use of HPCs for cell transplantation, three variables are essential: cell number, engraftment potential and differentiation state. The cell number administered may be critical for functional recovery of a damaged liver. An indication of the number necessary can be derived from hepatocyte transplantation studies. Jorns et al. provided a concise literature overview of hepatocyte transplantations in various species, including human, which may be most relevant for application in veterinary medicine [130]. The number of transplanted cells depends on the infusion rate and injection route, and can be divided over multiple sessions. It is accepted that for correction of a genetic metabolic disease, 2-5% repopulation is sufficient to correct the phenotype [13]. Generally, billions of hepatocytes are used for intraportal delivery in human. Engraftment potential of hepatocytes may be very different than that of stem cells. In addition, the host environment of the diseased liver, and thus the type of disease, determines successful engraftment and therefore the number of cells needed for functional recovery. Finally, the differentiation status of the HPCs is important for the success of transplantation. The stage of maturation may determine homing and engraftment ability of HPCs. For example, undifferentiated HPCs have the capacity to migrate [33,79]. On the other hand, a cell in a more differentiated state with developing hepatocyte characteristics might pose an attractive clinical application in cases of acute liver failure.

With respect to HPC transplantation, metabolic diseases will probably be the first to be addressed in both dog and human. In dogs, transplantation of hepatocytes has been reported in a number of studies, mostly in Dalmatians as a model for metabolic disease (hyperuricosuria) [131-133]. In these types of diseases, improvement of the phenotype can be accomplished by providing a relatively low number of cells from a healthy donor, or upon genetic correction of autologous cells. The COMMD1 deficient dog presenting with copper storage disease resulting in chronic hepatitis, provides an excellent model for clinical HPC transplantation trials [134,135]. Such studies will reveal important information on efficacy and safety of HPC transplantation and will facilitate translation of this therapeutic strategy to the veterinary and human clinic. Diseases with a more complex pathophysiology, such as chronic hepatitis involving fibrosis and remodeling of tissue architecture, will be more challenging. These types of diseases will require a multimodal approach targeting not only hepatocyte regeneration but also fibrosis resolution and modulation of inflammation. Current developments in anti-fibrotic therapies and the co-transplantation of mesenchymal stem cells or macrophages to modulate inflammatory responses may aid the development of new regenerative therapies for chronic and severe liver diseases in man and dog [136,137].

## Conclusions

**Figure 6:**
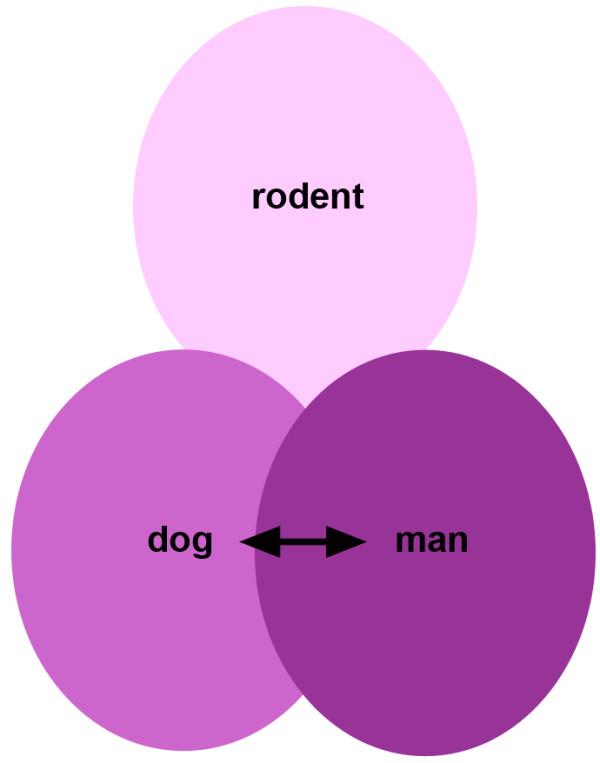
**Translational medicine in veterinary and human hepatology.** In HPC biology inter-species differences and similarities can be found. When reviewing HPC markers, niche characteristics and HPC response in health and disease, dogs share many similarities with man. Furthermore, dogs can bridge the gap between experimental rodent studies and human clinical application. Human medicine could benefit from its canine counterpart by appreciating the dog as a target species as well as a large animal model for the development of new therapies.

There is much promise in the use of HPCs in regenerative therapies for both human and veterinary medicine. Fundamental studies in toxic and genetic rodent models, together with (comparative) histo-pathological studies in humans have determined HPCs to be clinically relevant. In canines, important molecular and cellular reaction patterns in particular liver diseases are reported, and characterize HPCs and their niche. Overall, HPC marker expression in dogs is comparable to that of humans, as is response to injury and the cell types involved in modulating HPC response. This suggests that the therapeutic potential of these cells is similar in dog when compared to man, and opens up the potential for developing new strategies for currently untreatable canine liver diseases (Figure 6).

On the other hand, there is still much to be conducted in feline hepatology. As with canine investigations, studies on cat liver disease and pathology would benefit from focusing on the molecular mechanisms of disease and regeneration in comparison to human and canine models, including the presence and characteristics of feline HPCs. In addition, feline lipidosis and cholangitis, diseases that are rare in dogs, may provide important models for human steatohepatitis and biliary disease.

We conclude that humans and dogs share many similarities with respect to liver disease and HPC biology, especially since dogs have spontaneous liver disease that equally requires treatment. With the emergence of regenerative medicine, veterinary and human medicine have the unique opportunity to advance potential therapies and technologies together. In particular, human medicine could greatly benefit from HPC-based trials in dogs.

## Unpublished observations

Valtolina C, Muys J, Penning LC, Grinwis GG, Schotanus BA: Characterization of the feline hepatic progenitor cell niche in health and disease, manuscript in preparation.

Van Sprundel RGHM, Van Den Ingh TSGAM, Guscetti F, Kershaw O, Kanemoto H, Van Gils HM, Rothuizen J, Spee B: Classification of primary hepatic tumours in the cat, manuscript in preparation.

## Abbreviations

HPC: Hepatic progenitor cell; HCC: Hepatocellular carcinoma; PHx: Partial hepatectomy; HGF: Hepatocyte growth factor; PCNA: Proliferating cell nuclear antigen; DR: Ductular reaction; K (7): Keratin (7); ECM: Extracellular matrix; HSC: Hepatic stellate cell; MMP: Matrix metalloproteinase; TIMP: Tissue inhibitor of matrix metalloproteinase; αSMA: Alpha smooth muscle actin; NASH: Non-alcoholic steatohepatitis; NAFLD: Non-alcoholic fatty liver disease; LDH: Lobular dissecting hepatitis; CLC: Cholangiolocellular carcinoma.

## Competing interests

The authors declare that they have no competing interests.

## Authors’ contributions

Authors HS Kruitwagen, B Spee and BA Schotanus contributed equally to the writing of this manuscript and preparation of the figures. All authors read and approved the final manuscript.

## Authors’ information

Department of Clinical Sciences of Companion Animals, Faculty of Veterinary Medicine, Utrecht University, The Netherlands.
